# An efficient intrusion detection model based on convolutional spiking neural network

**DOI:** 10.1038/s41598-024-57691-x

**Published:** 2024-03-25

**Authors:** Zhen Wang, Fuad A. Ghaleb, Anazida Zainal, Maheyzah Md Siraj, Xing Lu

**Affiliations:** 1 Faculty of Computing, Universiti Teknologi Malaysia, Johor Bahru, 81310 Johor, Malaysia; 2 School of Data Science and Artificial Intelligence, Wenzhou University of Technology, Wenzhou, 325035 Zhejiang China; 3 College of Computing and Digital Technology, Birmingham City University, Birmingham, B47XG United Kingdom

**Keywords:** Spiking neural network, Convolutional neural network, Intrusion detection, Cyber security, Deep learning, Artificial intelligence, Computer science, Information technology

## Abstract

Many intrusion detection techniques have been developed to ensure that the target system can function properly under the established rules. With the booming Internet of Things (IoT) applications, the resource-constrained nature of its devices makes it urgent to explore lightweight and high-performance intrusion detection models. Recent years have seen a particularly active application of deep learning (DL) techniques. The spiking neural network (SNN), a type of artificial intelligence that is associated with sparse computations and inherent temporal dynamics, has been viewed as a potential candidate for the next generation of DL. It should be noted, however, that current research into SNNs has largely focused on scenarios where limited computational resources and insufficient power sources are not considered. Consequently, even state-of-the-art SNN solutions tend to be inefficient. In this paper, a lightweight and effective detection model is proposed. With the help of rational algorithm design, the model integrates the advantages of SNNs as well as convolutional neural networks (CNNs). In addition to reducing resource usage, it maintains a high level of classification accuracy. The proposed model was evaluated against some current state-of-the-art models using a comprehensive set of metrics. Based on the experimental results, the model demonstrated improved adaptability to environments with limited computational resources and energy sources.

## Introduction

Human society and Internet technology have become increasingly integrated. It is indisputable, however, that cyber-attacks are increasing for the current network environment^1,2^. Securing IoT systems is even more challenging. Energy, memory, communication, and computation power are often constrained on IoT devices and networks. Which makes them more vulnerable to cyberattacks. Cyber-attacks can cause serious damage, from financial losses to the disruption of critical services. Governments and organizations must take steps to ensure their systems are secure and their data is protected. To this end, the development and implementation of defense systems and strategies are necessary. Fortunately, security products for computers and networks are constantly evolving and expanding to ensure that they can adapt and reflect the risks they face. One of the most important products among all of these is intrusion detection systems (IDSs)^3^. An IDS monitors network traffic to detect suspicious activity and threats. Upon identifying potentially malicious activity, IDS alerts the IT manager to the possibility of a network intrusion. Since there is a large amount of network data available, the intrusion detection problem is well suited to DL methods^4^.

Artificial neural networks (ANNs) have been energized by a great deal of potential in the last decade, from multi-layer perceptron (MLP) in the first generation to deep neural networks (DNNs) in the second generation. Even with this great advancement, ANNs still lack the energy efficiency and online learning capabilities of biological neural networks^5^. Traditional deep learning models have been subjected to many attempts to reduce their power consumption. Numerous techniques have been developed to find more compact networks with similar performance fewer parameters and less complexity than the original network. These techniques include quantization^6^, pruning^7^, and knowledge distillation^8^. However, all these methods are just patching on top of the original and do not get to the root of the problem.

Though ANNs and DNNs are historically based on neural networks, they differ fundamentally in their structure, neural computations, and learning rules in comparison with biological neural networks^5^. In SNNs, the model closest to the biological neuron mechanism is used^9^. The SNN accumulates the input to the membrane voltage via the pulse neurons, and when the threshold has been reached, the pulse is then emitted to enable event-driven computations to take place. As a result of the sparse nature of pulse events and the event-driven manner in which they are computed, SNNs are capable of providing a higher level of energy efficiency^10^. Since SNNs have similar functional characteristics to biological neural networks, they can accommodate sparsity found in biological systems and are highly compatible with temporal codes^11^.

1. A time-value algorithm is proposed to encode the spike information triggered by the data. By using this encoding, complex spike trigger patterns can be mapped to a value that can be uniquely determined. Information can be provided more comprehensively to the processing processes without increasing model data transfer complexity.
2. A loss function is tailored to the proposed model. From metrics such as the accuracy of the current classification to the ranking of the correct classification in the inferred results, it can provide supervision to ensure the model approximates the correct result in several directions.
3. A dynamic thresholding strategy is developed for the model during gradient backpropagation. This is because the change in the amount of membrane potential in a neuron can reflect, to some extent, the degree of change in the input data. Therefore, in this paper, the slope of the fitted curve after cubic spline interpolation of this variable is used as the dynamic threshold for gradient in the subsequent backpropagation process.
4. A novel intrusion detection model based on SNNs and CNNs is proposed and implemented. It can be speculated that the model is capable of better adapting to resource-constrained environments and can continue to provide security to the target device in arduous conditions, based on the results of the experiments that have been conducted.

The remaining portions of this paper are organized as follows. A description of the related work is provided in “Related work”. In “Proposed scheme”, we describe our proposed solution. “Performance evaluation and discussion” evaluates the performance of the adopted models and discusses the results. In “Conclusions and future work”, the results of the experiments are summarized, and feasible directions for future research are suggested.

## Related work

Various types of models can be used for intrusion detection. In this paper, the focus is on the most relevant part, i.e., detection models associated with CNNs or SNNs. Of course, a comprehensive study of the model's performance is another important consideration. This section reviews the results of research in these directions in recent years and provides a summary of the status of these studies.

### CNN-based models

Deep learning algorithms are widely used in IDSs, and one of the most popular models is the CNN. Several studies have demonstrated that CNN models can be used to achieve good detection accuracy. In^12^, it recommends the use of a new CNN architecture type called mean convolution layer (CNN-MCL), which was developed for learning the content features of anomalies and identifying the anomaly. The CNN-MCL can be used in conjunction with an innovative form of convolutional layer that enables the education of low-level abnormal characteristics to design a strong network intrusion detection system. Testing the proposed model on the CICIDS2017 dataset produced favorable results regarding the detection of anomalies with high accuracy and low false alarm rates in comparison to other models. In^13^, using the minimum protocol information, field size, and offset, the authors propose the first preprocessing method, called "direct", for network IDS. Apart from direct preprocessing, they also propose two other techniques known as "weighted" and "compressed". Due to the requirement for additional network information, the direct conversion was compared with similar studies. In addition to direct, the proposed preprocessing methods are based on a field-to-pixel philosophy that exploits the convolutional features of each pixel to achieve the advantages of CNN. When evaluating the direct method, weighted and compressed conversion methods are used. As a result, the proposed direct preprocessing method coupled with a CNN produced a meaningful IDS in the NSL-KDD dataset. As opposed to focusing on broad categories of attacks, authors discuss various attacks within the same category^14^. DoS is different from other categories of KDD in that it has sufficient samples for training each attack. The authors also use CSE-CIC-IDS2018, which is one of the most recent IDS datasets. CSE-CIC-IDS2018 includes more sophisticated DoS attacks than KDD. Numerous experiments were conducted to determine the optimal CNN design for better performance. Evaluations of the performance of the models were conducted based on a comparison between CNNs and Recurrent Neural Networks (RNNs). It was proposed in the paper^15^ to use a fusion method of multi-convolutional neural networks (multi-CNN) to detect intrusions. Following the correlation, the feature data are divided into four parts, which are then converted into grayscale graphs based on the one-dimensional feature data. In the intrusion detection problem, CNN is introduced through the flow data visualization method, and the most effective of the four results is identified. As a result of the experiments, the multi-CNN fusion model was successfully demonstrated to provide a method for classifying the NSL-KDD dataset that is highly accurate and low in complexity. There were two models proposed by the authors^16^ based on deep learning for the classification of binary and multiclass network attacks. To develop our models, they use a convolutional neural network architecture. Moreover, a hybrid two-step preprocessing approach is presented to generate meaningful features. Feature engineering and dimensionality reduction are combined in the proposed approach. Two benchmark data sets are used to appraise the models' performance. A comparison is made between the performance of the proposed system and that of similar deep learning approaches published in the literature, as well as state-of-the-art classification models. Results from their experiments indicate that their models are accurate and recall well, outperforming similar models in the literature. A CNN intrusion detection model based on attention is proposed in the study^17^. The combination of the image generation methods presented in this paper results in a processing flow that is efficient and accurate. The experimental images were arranged according to the results of the importance analysis of the feature fields to optimize the use of the feature information in the experiments. As part of the process of building the detection model, a more integrated attention mechanism has been applied to CNN. On a subset of the CSE-CIC-IDS2018 dataset, a series of comparative experiments have been conducted, and the results indicate that the proposed detection process and model can rapidly complete the detection procedure while maintaining a high level of accuracy.

In addition, CNNs are often used in combination with other models to extract more features from the dataset, such as^18–24^. However, these models have the disadvantage of being significantly more complex and are not conducive to real-time, efficient data processing.

### SNN-based models

Current research related to SNN models is mainly focused on computer vision, such as image classification^25–27^ and object detection^28–30^, and there is little research for intrusion detection. The use of SNNs for intrusion detection is therefore of great importance to subsequent researchers.

The authors investigated the feasibility of using SNNs to detect cyberattacks in vehicles^31^. An autoencoder model is converted into spiking form to show exemplary results. Their comparison of SNN autoencoders with One-Class Support Vector Machines and Isolation Forests demonstrates that they outperform both models. The Gryphon advanced intelligence system is presented^32^. An evolving Spiking Neural Network One-Class Classifier (eSNN-OCC) is being used in the Gryphon System to detect unary anomalies in big industrial data. An advanced persistent threat (APT) is a type of cyberattack that is characterized by divergent behaviors and abnormalities. The machine learning algorithm corresponding to this algorithm can detect these abnormal behaviors and divergent behaviors very rapidly and efficiently. IDS-SNNDT is a new intrusion detection system that is based on spike neural networks and decision trees^33^. To reduce latency and minimize device power consumption, the non-leaky integrate neurons fire (NLIF) model was used in the SNN to select the optimal samples for input. To detect cyber-attacks, Rand order code (ROC) is also used with SNN. Based on three performance metrics: detection accuracy, latency, and energy consumption, the proposed method is compared with two other methods: IDS-DNN and IDS-SNNTLF. Simulation results indicate that the IDS-SNNDT method uses less power and has lower latency than IDS-DNN and IDS-SNNTLF. To analyze the input–output expressions of both leaky and nonleaky neurons, they consider a general class of single-spike temporal-coded integrate-and-fire neurons^34^. Using leaky neurons, authors show that SNNs are prone to overly nonlinear and complex input–output responses, which is a major cause of their difficulty in training and poor performance. In contrast to the widely held belief that spikes cannot be differentiated; this reason is more fundamental. In support of this claim, they demonstrate that SNNs built with nonleaky neurons can exhibit a simpler input–output response that is less complex and nonlinear. It has been demonstrated that SNNs can easily be trained and can perform better than other algorithms, as evidenced by experiments conducted with the SNNs over two popular datasets for network intrusion detection, the NSL-KDD, and the AWID. Based on their experiments, they demonstrate that the proposed SNNs outperform a comprehensive list of DNN models as well as classic machine learning models. According to this study, SNNs are both promising and competitive, contrary to the belief of many. Although many researchers have claimed that SNNs can improve performance in a wide range of areas, current research is not sufficiently exploiting these benefits.

Additionally, SNNs can be applied to relevant nonlinear regression studies. An integrated-and-fire neural network architecture combined with delays is presented to approximate real-valued function mappings within a specified degree of accuracy by using spiking neural networks^35^. An explicit numerical scheme based on the Spiking Neural Network (SNN) has been proposed to integrate time-dependent ordinary and partial differential equations (ODEs and PDEs) in a long time period^36^. A spike-encoded initial condition can be used to compute the solution at future timesteps after the network has been trained as an explicit numerical scheme. In this study^37^, an artificial intelligence algorithm is presented that can be applied to Engineering Mechanics Boundary Value Problems via neural computing. To calculate the nonlinear (physically and geometrically) response of shock wave-loaded plate elements, they propose a hybrid model combining the Legendre Memory Unit (LMU) with spiking recurrent cells and classical dense transformations.

### Performance studies of models

Despite the numerous studies claiming that SNNs are efficient^38–40^, a lot of effort is still needed to exploit this efficient performance of them. In comparison, a human brain operates within a power budget of approximately 20 watts, while artificial neural networks incur huge costs in terms of processing power, memory performance, and energy consumption^41^. This could also indicate that current neural network models still have a lot of untapped potential.

To enhance the storage and computing efficiency of TSR, the authors propose a hybrid SNN-CNN network with weights implemented in RRAM^42^. Comparing the SNN-CNN hybrid network with state-of-the-art CNN methods, the hybrid network achieves similar accuracy with 69.21% less weighted parameters and 81.55% lower power consumption. Using the Differentiation on Spike Representation (DSR) method, high performance comparable to ANNs is achieved with minimal latency^43^. The study shows that on both static and neuromorphic datasets, DSR can achieve state-of-the-art SNN performance with low latency. SNNs are utilized to build an ultra-low-power radio frequency fingerprinting identification (RFFI) system^44^. Spiking neurons are optimized so that high accuracy is achieved with very few spikes. Additionally, attention mechanisms are utilized to further improve RFFI performance by addressing signals in multiple dimensions. In comparison to ANNs of comparable accuracy, the SNN-based RFFI system consumes 64% less power. It is proposed that Activation Consistently Coupled ANN-SNNs (${AC}^{2}AS$) can be trained in a fast and memory-efficient manner^45^. To reduce the occurrence of noisy spikes, the researchers designed an adaptive threshold adjustment algorithm (ATA). Experiments show that their (${AC}^{2}AS$)-based models exhibit good performance on benchmark datasets. An adaptive threshold mechanism has been proposed for improving the balance between the weight and threshold of SNNs by analyzing the differences between analog neurons and spiking neurons^46^. On CIFAR10, this mechanism outperformed most of the recently proposed SNNs in terms of accuracy, accuracy loss, and network latency, and achieved state-of-the-art results on CIFAR100. It is proposed to employ a Dynamic Threshold Integrate and Fire (DTIF) model that exploits the variability in thresholds of biological neurons to increase spike activity^47^. To reduce latency, the threshold is dynamically adjusted at each simulation time step to increase spike activity. In contrast to state-of-the-art conversion methods, the ANN-to-SNN conversion using the DTIF model offers lower latency and competitive image classification accuracy. The findings of these studies also provide insights into ways in which energy-efficient models may be designed^48–50^. In summary, current research is focused on the following aspects. One is in the training phase to minimize the cost of model training. Several others are in the validation phase, improving model accuracy, execution efficiency, and energy consumption metrics.

## Proposed scheme

A lightweight efficient intrusion detection model based on convolutional spiking neural networks is proposed in this paper. To achieve excellent results in terms of processing performance, we must exploit the strengths of both spiking neural networks and convolutional neural networks throughout the process of creating the processing framework. Of course, the complexity of the model has been reduced as much as possible to allow the final model to achieve the goal of working accurately and efficiently even in environments where computational resources are limited. The design of the entire model was a result of this balance between the need for simplicity and efficiency and the need for accuracy.

### Data pre-processing

**Figure 1:**
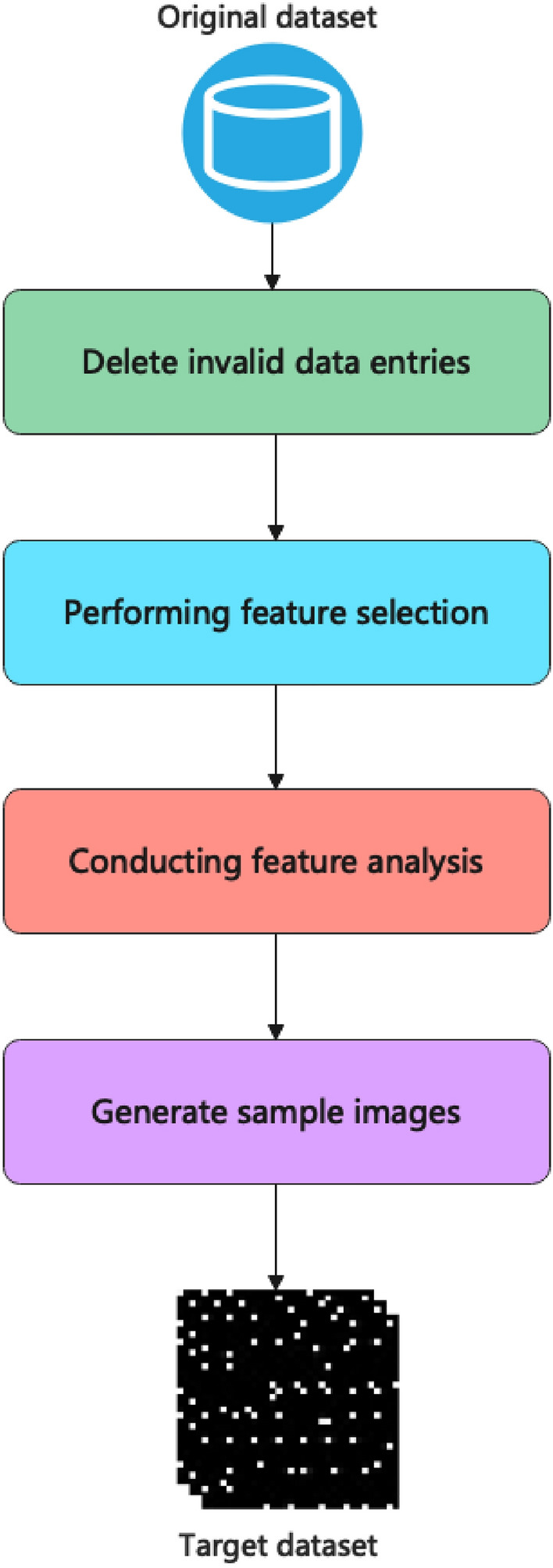
Data pre-processing.

The data samples used during the experiments were constructed based on CSE-CIC-IDS2018^51^ and CIC-DDoS2019^52^, respectively. These two datasets consist of information about packets captured on the network. Each sample consists of features extracted from a network data packet used to distinguish between different data types. The construction process of the experimental samples is shown in Fig. 1. Since neither software nor hardware can handle all exception scenarios effectively, a small number of invalid field values will have to be generated. To counter this, data entries containing invalid field values are removed from the dataset. Then remove fields that are not relevant to the specific classification: such as source port, source IP address, timestamp, etc. A further reduction in computational costs and complexity was achieved by filtering 64 features using the SelectKBest method in the Sklearn library. Thereafter it is necessary to develop appropriate coding rules by analyzing each feature on a case-by-case basis.

For each feature field, for which information is extracted, 16 bits of space will be allocated for storing the results. Each feature is represented as a binary matrix in the form of a 4 × 4 matrix. In total, 64 features are selected (arranged 8 × 8), which will be represented by a 32 × 32 matrix^17^.

Some fields correspond to fewer instances of taking values that are encoded directly using one-hot coding; for fields with integer discrete values with a moderate range, encode its value in binary; With discrete-value fields in a wide range, the values are sorted in ascending order first, and then the original value is replaced with the sorted number. It is equivalent to translating these values and unifying their differences. An analysis of outliers is then performed on these fields, which have a wide range of discrete and continuous values. These values that are determined to be outliers are replaced by the nearest normal values. Then each of these fields will be scaled to [0, 100]. The scaled values were then one-hot encoded. In other words, when the value reaches a certain level, the binary bit corresponding to the level changes to 1, while all other bits are zeros.

After these processes, the network traffic data information will be converted into a binary matrix of 0 and 1 elements. It is like doing a spike calculation operation on text info and getting their corresponding spikes. To facilitate categorical storage and visual observation, these matrix elements will be uniformly multiplied by 255, and then each matrix will be converted to a greyscale image. During the experiments, two datasets, CSE-CIC-IDS2018 and CIC-DDoS2019, were used with 6 and 10 data types, respectively. For clarity and efficiency, 5000 samples of each type will be selected for subsequent experiments in the order in which they appear in the dataset.

### Spiking neuron model

In a SNN, the most basic functional unit is the spiking neuron. Each layer of the SNN has one or more neurons. Information is processed within a certain window of time by these neurons. Assume that $t$ is the current time window. Then, each neuron will have $t$ chances to calculate the recharge potential based on the input data and attempt to generate spiking. All neurons began with a membrane potential of zero. The membrane potential of the $i$th neuron is updated at every time step in the following manner:

1 $${V}_{i}\left(t\right)= {V}_{i}\left(t-1\right)+{I}_{i}(t-1)$$

${V}_{i}\left(t\right)$ and ${V}_{i}\left(t-1\right)$ denote the membrane potentials of the $i$th neuron at time step $t$ and, $t-1$ respectively. Calculated from the input and connection weights, ${I}_{i}(t-1)$ represents the increment in membrane potential at time step $t-1$.

2 $${I}_{i}(t-1) = {\sum }_{j}{W}_{ji}{J}_{j}(t-1)$$

where ${W}_{ji}$ denotes the weights connected to the $i$th neuron; ${J}_{j}(t-1)$ denotes the value passed to $i$th neuron from the $j$th input of the previous layer at time step $t-1$.

A spike is generated when ${V}_{i}$ exceeds its threshold, ${V}_{thr}$, and ${V}_{i}$ is reset:

3 $${V}_{i}\left(t\right)= {(V}_{i}\left(t\right)- {V}_{thr})* \alpha \,and \,{S}_{i}(t)=1, if \,{V}_{i}\left(t\right)>{V}_{thr}$$

Here $\alpha \in (0, 1)$ denotes the attenuation factor. That is, when a spike is triggered, the potential of ${V}_{i}$ is reset to a value that is the product of the portion of the spike threshold that is exceeded and $\alpha$. Here, to distinguish the difference in membrane potentials held by the neurons during excitation of the spike signal, the reset was not uniformly set to 0. It will then be possible for the potential built up during the previous spike to play a role in triggering the spike when it occurs next. ${S}_{i}(t)$ indicates the spike triggering of the $i$th neuron at the time step $t$. A value of 1 means that it is triggered, and the default is 0. The spiking information generated by these neurons in the time window t will be further encoded and provided as input information to the next neural network layer. To put it another way, the sequence of 0's and 1's obtained from each neuron in chronological order is encoded using some form of rule and transmitted to the next layer of the neural network. Coding methods such as rate coding and temporal coding are widely and commonly used.

### Detection model

**Figure 2:**
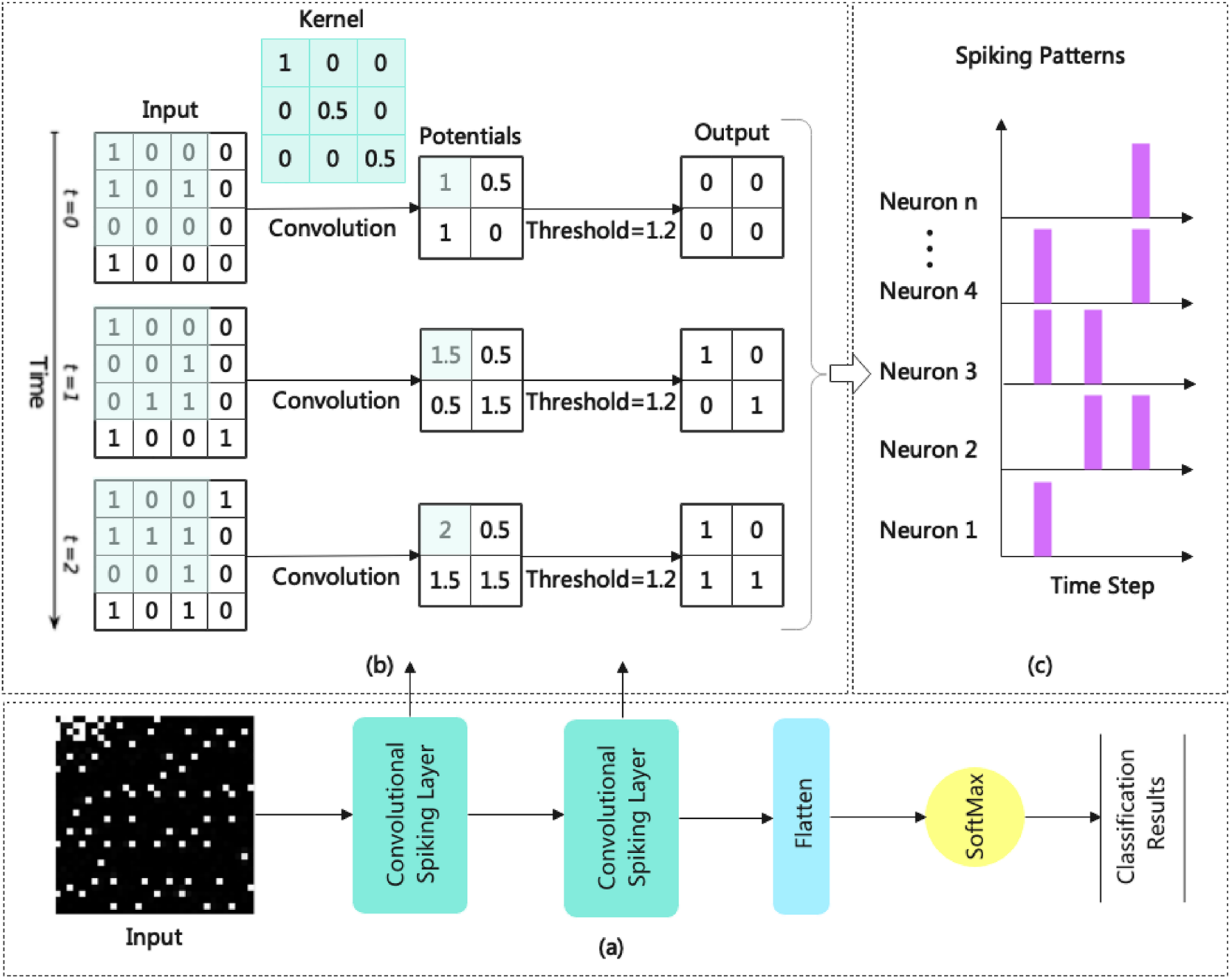
An example architecture of the proposed convolutional spiking neural network. (**a**) An overview of the functional layers in the model and how they are organized; (**b**) the computational procedure for the convolutional spiking layer is explained. In the example, the convolution kernel size is 3*3, the step size is 1, the spiking threshold is 1.2 and time window is 3; (**c**) demonstrates the spiking patterns exhibited by the neurons during computation. Based on these patterns it is possible to classify the data currently being processed.

In Fig. 2, an example architecture of the proposed convolutional spiking neural network is shown with two convolutional spiking layers. Depending on the desired recognition task, the architectural properties of a network (e.g., the number of layers and receptive field sizes) as well as learning parameters should be optimized.

The convolutional spiking layer is the model's primary functional layer, which contains both convolutional computation and spike triggering. Equation (1) illustrates the logic of the convolution calculation.

4 $$y=\sigma (K\odot x+b)$$

Here $x$ is the values of the input, e.g. a part of the matrix; $K$ denotes the convolution kernel; $\odot$ denotes the operation of multiplying the elements of two matrices in the same position and accumulating their products. $b$ stands for bias and can be set to 0 if not required. $\sigma$ is then the activation function, e.g. sigmoid, applied to the above calculation, but it also can be no operation. The internal potential of the $i$th neuron is updated at every time step in the following manner:

5 $${V}_{i}\left(t\right)= {V}_{i}\left(t-1\right)+y$$

${V}_{i}\left(t\right)$ and ${V}_{i}\left(t-1\right)$ denote the internal potentials of the $i$th neuron at moments $t$ and $t-1$, respectively. As soon as the membrane potential has been updated, determine whether it is time to trigger the spike signal and how to reset the membrane potential once the spike signal has been triggered as described in Eq. (3).

The signal associated with the last convolutional spiking layer will be encoded using the "time-value", where spikes triggered first will be considered to have a higher value than spikes triggered later. The specific rules are shown in Algorithm 1.

**Figure:**
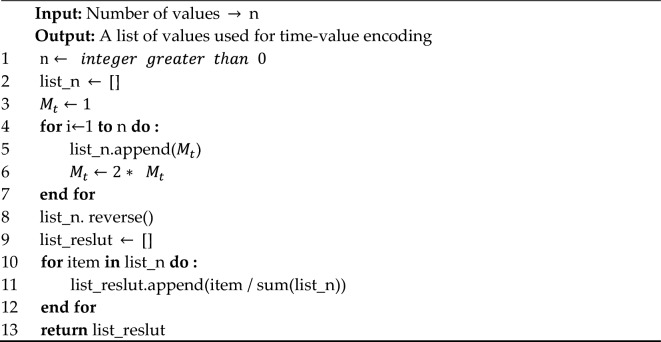
**Algorithm 1**: Time-value encoding for the last convolutional spiking layer.

The main calculation logic involved is shown in the following equation:

6 $$\left\{\begin{array}{c}{M}_{t}=2* {M}_{t+1} , if \,mt=max(t), {M}_{mt}=1\\ {P}_{t}= \frac{{M}_{t}}{{\sum }_{j}{M}_{j}}\end{array}\right.$$

As can be seen, the last occurrence of ${M}_{mt}$ is the smallest, and its value can be considered the unit 1. Forward moments correspond to 2 times the value of the subsequent moment. The value of ${P}_{t}$ is the value that can be obtained by generating a spike at moment $t$. That is, it represents the proportion of ${M}_{t}$ in the total sum of ${M}_{j}$. Clearly, $\sum {P}_{t}$ is convergent and does not change as the time window floats.

For better training, a loss function is also designed for the proposed model. Algorithm 2 illustrates its main logic. As shown in the algorithm, the final loss value is mainly determined by two factors, $\alpha$ and $\beta$. $\alpha$ factor indicates the difference between the type predicted by the model and the correct type. $\alpha$ is scaled up by multiplying it by the number of classifications, n_class, since a higher number of classifications tends to result in smaller values. The task of the $\beta$-indicator is to haul the probability of correct classification towards its maximum value. It also takes into account the ranking of the correct classification in the model prediction results. The reason for adding 1 to $ids$ is that the ranking starts at 0. Even if the current ranking is 0 (i.e. first place), as long as its probability value has not reached its maximum value, it means that there is still room for optimization.

**Figure:**
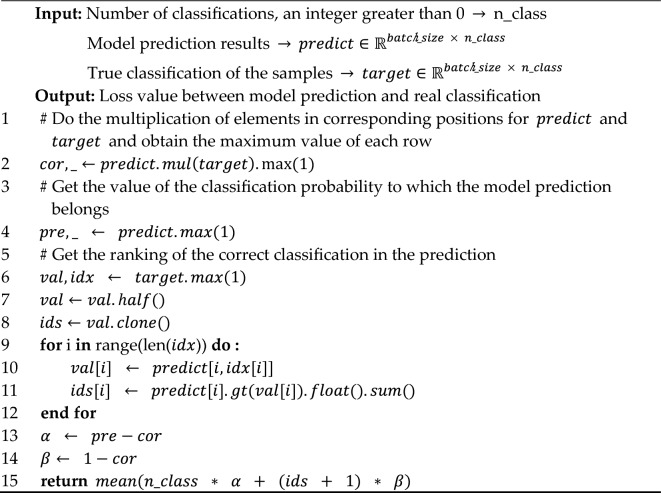
**Algorithm 2**: Loss function designed for the proposed model.

For better results when using gradient backward propagation for model training, gradient screening is also required. Due to the discrete nature of spike signals, it is not directly possible to determine their gradient values. When gradients with too large absolute values are used for model parameter tuning, they are prone to causing perturbations that interfere with the training process. Therefore, it is common to limit the gradient range when backpropagating. A fixed threshold can only be determined by considering the whole dataset. Dynamic thresholds can, on the other hand, be adjusted for each batch of training data. This results in a finer adjustment of the gradient range, allowing the model to be more flexible in finding optimal results in the solution space. Cubic spline interpolation^53^ is used to curve fit the amount of membrane potentials in neurons. The slope of the corresponding position is used as the final threshold for gradient back propagation. This is because the change in the amount of membrane potential in a neuron can reflect, to some extent, the degree of change in the input data. Basically, the algorithm fits a piecewise function in the form of:

7 $$S\left(x\right)=\left\{\begin{array}{c}{s}_{1}\left(x\right), if \,{x}_{1}\le x<{x}_{2}\\ {s}_{2}\left(x\right), if \,{x}_{2}\le x<{x}_{3}\\ \dots \\ {s}_{n-1}\left(x\right), if \,{x}_{n-1}\le x<{x}_{n}\end{array}\right.$$

Here ${s}_{i}$ is a polynomial of third degree defined as follows:

8 $$s_{i} \left( x \right) = a_{i} \left( {x - x_{i} } \right)^{3} + b_{i} \left( {x - x_{i} } \right)^{2} + c_{i} \left( {x - x_{i} } \right) + d_{i} \,\quad {\text{for}} \,i = 1,2, \ldots ,n - 1$$

In this process, it is essential for the first and second derivatives of these n-1 equations to be known, and they are as follows:

9 $${s}_{i}^{\prime}\left(x\right)={{3a}_{i}(x-{x}_{i})}^{2}+{2b}_{i}(x-{x}_{i})+{c}_{i}$$

10 $$s^{\prime\prime}_{i} \left( x \right) = 6a_{i} \left( {x - x_{i} } \right) + 2b_{i} ,\quad {\text{for}} \,i = 1,2, \ldots ,n - 1$$

The spline needs to meet the following qualifications:

- The interpolation will be performed on all data points by S(x).
- During the interval $[{x}_{1},{x}_{n}] S(x)$ will be continuous.
- During the interval $[{x}_{1},{x}_{n}] {S}^{\prime}(x)$ will be continuous.
- During the interval $[{x}_{1},{x}_{n}] {S}^{{\prime}{\prime}}(x)$ will be continuous.

The curve to be fitted $S(x)$ can be determined based on these constraints. After calculating the slope of the corresponding point on $S(x)$, the gradient backpropagation threshold can be identified. The calculated slope can be normalized to ensure that the threshold is within a reasonable range. Several experiments have found that computing the slope only from one of a batch of training samples mitigates overfitting and reduces computational complexity.

During model training, the learning rate is adjusted with the following strategy:

11 $$\left\{\begin{array}{c}{\eta }_{1}=0.001\\ {\eta }_{t}= 0.5*{\eta }_{1}\left(1+{\text{cos}}\left(\frac{t}{{T}_{{\text{max}}}}\pi \right)\right), 2\le t \le {T}_{{\text{max}}}\end{array}\right.$$

${\eta }_{1}$ is the initial learning rate set by the model during the first epoch of training. ${T}_{{\text{max}}}$ is the total number of epochs the model has to be trained for and $t$ is the current number of training rounds.

### Evaluation metrics

In the experiments, each model was evaluated in several dimensions, and the main evaluation criteria are as follows:

(1) Detection accuracy, which measures a model's basic capability. In this study, the following evaluation indicators were used:

12 $$\mathrm{True \,Positive \,Rate}= \frac{{\text{TP}}}{{\text{TP}}+{\text{FN}}}$$

13 $$\mathrm{False \,Positive \,Rate}= \frac{{\text{FP}}}{{\text{FP}}+{\text{TN}}}$$

14 $${\text{Accuracy}}= \frac{{\text{TP}}+TN}{{\text{TP}}+{\text{FP}}+{\text{TN}}+{\text{FN}}}$$

15 $${\text{Precision}}= \frac{{\text{TP}}}{{\text{TP}}+{\text{FP}}}$$

16 $${\text{F}}1-{\text{score}}= \frac{2 \times \mathrm{ Precision }\times \mathrm{ True \,Positive \,Rate}}{{\text{Precision}}+\mathrm{True \,Positive \,Rate}}$$

A true positive is indicated by the letter TP. A true negative is indicated by the letter TN. False positives and false negatives are indicated by the letters FP and FN, respectively.

(2) Model complexity is characterized by the number of parameters and computation required.

(3) Execution speed, as measured by the number of samples processed every second.

(4) Energy consumption is calculated as the average power consumption per 10,000 samples.

As a result of these metrics, a more comprehensive picture of the overall performance of the model can be obtained concerning accuracy, computational resource consumption, energy consumption, etc.

The efficiency metrics are demonstrated in Table 1. Energy consumption here is a count of the model's electrical energy consumption after processing 10,000 samples. Samples/s quantifies the rate at which the model processes experimental samples. The number of parameters of the model is mainly indicative of the complexity of the model. Floating-point operations (FLOPs) indicate the amount of floating-point computation required by the model. Model size reflects the amount of space occupied by the model after training is complete. This metric directly affects the model's space footprint at storage and runtime. The experimental setting for obtaining these data is as follows:

- Operating system: Linux-5.15.120 + -x86_64-with-glibc2.31.
- CPU: Intel(R) Xeon(R) @ 2.20 GHz, 4 Core(s), 120.00 W.
- RAM: 32 GB, 11.76 W.

**Table 1:** Efficiency metrics.

Model	Energy consumption (kWh $\times {10}^{-4}$)	Samples/s	Parameters	FLOPs	Model size (MB)
CIFARNet-GLIF	277.06228	6	45,028,352	4,972,892,160	180.16
ResNet18-LGLIF	368.12367	4	11,175,239	7,048,941,568	44.87
ResNet18-GLIF	373.16147	4	11,346,304	7,049,455,616	45.55
SpikeDHS	631.76467	6	14,777,148	11,987,675,136	183.88
SpikingGCN	19.56754	74	273,130	3,156,111,360	1.1
NAS-SNN	1455.09684	3	44,023,140	24,891,346,944	364.92
Spikformer	179.32850	9	9,330,010	3,743,420,160	37.54
CNN	8.19181	264	17,189,450	149,492,736	68.77
Our model	1.77497	5333	7482	204,800	0.03363

A GPU was used for acceleration during the comparison models training. This GPU was configured as follows:

- NVIDIA Tesla P100 (16 GB) GPU.

## Performance evaluation and discussion

For evaluation, the following comparison models were used in the experiments.

GLIF^54^ is a unified spiking neuron that fuses different bio-features in different neuronal behaviors, expanding the representation space of spiking neurons. It is possible to learn gate factors in GLIF during training, which determine the proportion of fused bio-features. By combining all learnable membrane-related parameters, this method can generate spiking neurons that are constantly changing, increasing their heterogeneity as well as their adaptability.

- CIFARNet-GLIF^54^: CIFARNet uses GLIF neurons.
- ResNet18-LGLIF^54^: ResNet18 uses GLIF with a layer-wise parameter-sharing scheme.
- ResNet18-GLIF^54^: ResNet18 uses GLIF neurons.
- SpikeDHS^55^: The spike-based computation is performed not only at the cell level but also at the layer level.
- SpikingGCN^56^: A framework that integrates the embedding of Graph Convolutional Networks (GCNs) with the bio fidelity characteristics of SNNs ends-to-end.
- NAS-SNN^57^: As in recent NAS approaches, this algorithm selects an architecture that represents diverse spike activation patterns across different data samples without training.
- Spikformer^58^: Based on leveraging the self-attention capabilities and biological properties of SNNs, a novel Spiking Self-Attention (SSA) algorithmic framework is developed.
- CNN^59^: Optimized convolutional neural network model.

The correspondence of data types and category numbers in dataset CSE-CIC-IDS2018, from 0 to 5, is Benign, DoS, DDoS, Botnet, Infiltration, and Brute Force. The data types represented by the classifications 0 to 9 in the CIC-DDoS2019 dataset are Benign, DrDoS_SNMP, TFTP, DrDoS_UDP, DrDoS_NetBIOS, DrDoS_MSSQL, Syn, DrDoS_SSDP, DrDoS_DNS, and UDP-lag, respectively.

After experimenting with layers between 1 and 5, it was ultimately determined that two layers provide the best combination of accuracy and efficiency of the spiking neural network. Therefore, the proposed model consists of 2 convolutional spiking layers and 1 linear layer. The time window for these 2 convolutional spiking layers was set to 4. The batch size for training is 40. Where the input shape of the first layer is (40, 1, 32, 32). These four values indicate the batch size, the number of channels, and the number of rows and columns in the input matrix. The convolutional kernel size of the layer is 4*4, the step size is 4 and the padding strategy is “valid”. The output shape of the first layer is (40, 16, 8, 8) as is the input shape of the second layer. The second layer convolution kernel size is 2*2, step size is 2 and padding strategy is “valid”. The output shape of the second layer is (40, 32, 4, 4). The number of neurons per layer is determined according to the minimum value of the input shape. As the output of the convolutional spiking network layer consists of a sequence of 0 s and 1 s that is encoded by the "time-value" algorithm at the end, no other regularization operations are applied. The preset spike trigger threshold is 0.3. The initial learning rate during training was 0.001. The total number of training rounds is 50. According to Eq. (11), the learning rate per round is adjusted.

The remarkable advantages of the proposed model can be seen in the experimental results. The first metric records the power consumption of the model after processing 10,000 samples. The consumption of energy is particularly important for equipment that has a limited supply of energy (e.g., devices using mobile power or batteries) since energy indicates the equipment's long-term viability. It is evident from the experimental results that the proposed model can reduce energy consumption at least by 70% or more compared to the other models, and even by more than 90% compared to the energy-consuming model. For the same amount of power supply, the model proposed in the paper can benefit from lower energy consumption. This will enable the host equipment to operate for a longer period, while providing more durable protection.

The Samples/s indicator reflects the efficiency of the model in processing samples, which mainly reflects the response speed of the model. More samples processed per second indicate a quicker response time and the ability to provide timely feedback regarding potential risks. As can be seen from the above results, the model proposed in this paper is at least 20 times faster than other models. It is even more than 500 times faster than most models.

The size of the number of parameters of the model is an important reference for the complexity of the model. Since most of the parameters are required to be trained to fit the target dataset. Therefore, the cost of the model in the training process, such as computational resources and time, can be largely reflected in this metric. In this regard, the proposed model still has a significant advantage. This means that the model can complete training and validation faster. This feature is especially important during the model development phase, allowing designers to put their ideas into practice and get feedback sooner.

FLOPs are mainly concerned with how much computation was performed by the model during the sample processing. It is a good reflection of the occupancy of computational units by the corresponding model in processing the samples. The fewer computing units the model needs to occupy, the less impact it will have on the original functionality of the device. Especially for computing resource-poor devices, adding new applications may even lead to intermittent failure of its original functionality. It is therefore necessary to reduce this indicator as much as possible, which is more conducive to maintaining the proper functioning of the original function. In Table 1, shows the total amount of computation required by the model to process one sample. In this respect, the proposed model still has a notable advantage. Compared to other models, it can be reduced by more than 90%.

The proposed model also has an obvious advantage as far as the indicator of model size is concerned. The data presented in the above table is the space required to be occupied in the storage medium after the model has been trained. For a model to provide protection on a target device, it necessarily requires the device to be able to store, load and run the model. The smaller the metric, the lower the corresponding model's demand for storage resources. Other models with even the smallest volume are more than 30 times larger than the model being proposed. With this feature, the proposed model has the potential to better accommodate storage-poor devices.

Of course, it is not enough to focus only on efficiency metrics; comparing efficiency among them needs to be based on the same level of correctness. Table 2 demonstrates the multi-classification accuracy of these models in different datasets.

**Table 2:** Classification accuracy.

Model	CSE-CIC-IDS2018	CIC-DDoS2019
CIFARNet-GLIF	98.82	99.86
ResNet18-LGLIF	98.82	99.88
ResNet18-GLIF	98.77	99.86
SpikeDHS	98.78	99.89
SpikingGCN	98.75	99.86
NAS-SNN	90.82	98.08
Spikformer	98.67	99.64
CNN	**98.88**	**99.90**
Our model	98.82	99.86

Significant values are in bold.

Where Tables 3, 4, 5, 6, 7, 8, 9, 10 and 11 and Figs. 3, 4, 5, 6, 7, 8, 9, 10 and 11 show the detailed experimental results for each model, respectively.

**Table 3:** CIFARNet-GLIF validation.

Category	True positive rate		False positive rate		Accuracy		Precision		F1-score	
	IDS-2018	DDoS-2019	IDS-2018	DDoS-2019	IDS-2018	DDoS-2019	IDS-2018	DDoS-2019	IDS-2018	DDoS-2019
0	0.985	0.999	0.011	0.000	0.998	1.000	0.95	1.00	0.97	1.00
1	0.999	1.000	0.000	0.000	1.000	1.000	1.00	1.00	1.00	1.00
2	1.000	1.000	0.000	0.000	1.000	1.000	1.00	1.00	1.00	1.00
3	1.000	1.000	0.000	0.000	1.000	1.000	1.00	1.00	1.00	1.00
4	1.000	0.998	0.000	0.000	1.000	1.000	1.00	1.00	1.00	1.00
5	0.944	0.997	0.003	0.000	0.998	0.999	0.98	1.00	0.96	1.00
6	–	0.999	–	0.000	–	1.000	–	1.00	–	1.00
7	–	0.995	–	0.000	–	0.999	–	1.00	–	1.00
8	–	0.999	–	0.000	–	1.000	–	1.00	–	1.00
9	–	0.999	–	0.000	–	1.000	–	1.00	–	1.00

**Table 4:** ResNet18-LGLIF validation.

Category	True positive rate		False positive rate		Accuracy		Precision		F1-score	
	IDS-2018	DDoS-2019	IDS-2018	DDoS-2019	IDS-2018	DDoS-2019	IDS-2018	DDoS-2019	IDS-2018	DDoS-2019
0	0.984	0.999	0.011	0.000	0.988	1.000	0.95	1.00	0.97	1.00
1	0.999	1.000	0.000	0.000	1.000	1.000	1.00	1.00	1.00	1.00
2	1.000	1.000	0.000	0.000	1.000	1.000	1.00	1.00	1.00	1.00
3	1.000	1.000	0.000	0.000	1.000	1.000	1.00	1.00	1.00	1.00
4	1.000	0.998	0.000	0.000	1.000	1.000	1.00	1.00	1.00	1.00
5	0.946	0.997	0.004	0.000	0.988	0.999	0.98	1.00	0.96	1.00
6	–	1.000	–	0.000	–	1.000	–	1.00	–	1.00
7	–	0.994	–	0.000	–	0.999	–	1.00	–	1.00
8	–	1.000	–	0.000	–	1.000	–	1.00	–	1.00
9	–	1.000	–	0.000	–	1.000	–	1.00	–	1.00

**Table 5:** ResNet18-GLIF validation.

Category	True positive rate		False positive rate		Accuracy		Precision		F1-score	
	IDS-2018	DDoS-2019	IDS-2018	DDoS-2019	IDS-2018	DDoS-2019	IDS-2018	DDoS-2019	IDS-2018	DDoS-2019
0	0.981	1.000	0.011	0.000	0.988	1.000	0.95	1.00	0.97	1.00
1	0.999	1.000	0.000	0.000	1.000	1.000	1.00	1.00	1.00	1.00
2	1.000	1.000	0.000	0.000	1.000	1.000	1.00	1.00	1.00	1.00
3	1.000	1.000	0.000	0.000	1.000	1.000	1.00	1.00	1.00	1.00
4	1.000	0.997	0.000	0.000	1.000	1.000	1.00	1.00	1.00	1.00
5	0.946	0.995	0.004	0.000	0.988	0.999	0.98	1.00	0.96	1.00
6	–	1.000	–	0.000	–	1.000	–	1.00	–	1.00
7	–	0.995	–	0.000	–	0.999	–	1.00	–	1.00
8	–	0.999	–	0.000	–	1.000	–	1.00	–	1.00
9	–	1.000	–	0.000	–	1.000	–	1.00	–	1.00

**Table 6:** SpikeDHS validation.

Category	True positive rate		False positive rate		Accuracy		Precision		F1-score	
	IDS-2018	DDoS-2019	IDS-2018	DDoS-2019	IDS-2018	DDoS-2019	IDS-2018	DDoS-2019	IDS-2018	DDoS-2019
0	0.989	1.000	0.012	0.000	0.988	1.000	0.94	1.00	0.97	1.00
1	0.999	1.000	0.000	0.000	1.000	1.000	1.00	1.00	1.00	1.00
2	1.000	1.000	0.000	0.000	1.000	1.000	1.00	1.00	1.00	1.00
3	1.000	1.000	0.000	0.000	1.000	1.000	1.00	1.00	1.00	1.00
4	1.000	0.998	0.000	0.000	1.000	1.000	1.00	1.00	1.00	1.00
5	0.938	0.997	0.002	0.000	0.988	0.999	0.99	1.00	0.96	1.00
6	–	1.000	–	0.000	–	1.000	–	1.00	–	1.00
7	–	0.994	–	0.000	–	0.999	–	1.00	–	1.00
8	–	1.000	–	0.000	–	1.000	–	1.00	–	1.00
9	–	1.000	–	0.000	–	1.000	–	1.00	–	1.00

**Table 7:** SpikingGCN validation.

Category	True positive rate		False positive rate		Accuracy		Precision		F1-score	
	IDS-2018	DDoS-2019	IDS-2018	DDoS-2019	IDS-2018	DDoS-2019	IDS-2018	DDoS-2019	IDS-2018	DDoS-2019
0	0.980	1.000	0.011	0.000	0.988	1.000	0.95	1.00	0.96	1.00
1	0.999	1.000	0.000	0.000	1.000	1.000	1.00	1.00	1.00	1.00
2	1.000	1.000	0.000	0.000	1.000	1.000	1.00	1.00	1.00	1.00
3	1.000	1.000	0.000	0.000	1.000	1.000	1.00	1.00	1.00	1.00
4	1.000	0.999	0.000	0.000	1.000	1.000	1.00	1.00	1.00	1.00
5	0.946	0.993	0.004	0.000	0.988	0.999	0.98	1.00	0.96	1.00
6	–	1.000	–	0.000	–	1.000	–	1.00	–	1.00
7	–	0.995	–	0.001	–	0.999	–	0.99	–	0.99
8	–	0.999	–	0.000	–	1.000	–	1.00	–	1.00
9	–	1.000	–	0.000	–	1.000	–	1.00	–	1.00

**Table 8:** NAS-SNN validation.

Category	True positive rate		False positive rate		Accuracy		Precision		F1-score	
	IDS-2018	DDoS-2019	IDS-2018	DDoS-2019	IDS-2018	DDoS-2019	IDS-2018	DDoS-2019	IDS-2018	DDoS-2019
0	0.913	0.998	0.078	0.000	0.920	1.000	0.71	1.00	0.80	1.00
1	0.945	1.000	0.000	0.000	0.992	1.000	1.00	1.00	0.97	1.00
2	1.000	1.000	0.001	0.000	1.000	1.000	1.00	1.00	1.00	1.00
3	1.000	1.000	0.002	0.000	0.998	1.000	0.99	1.00	1.00	1.00
4	0.981	0.998	0.001	0.000	0.997	1.000	1.00	1.00	0.99	1.00
5	0.607	0.833	0.029	0.001	0.913	0.982	0.80	0.99	0.69	0.91
6	–	0.998	–	0.000	–	1.000	–	1.00	–	1.00
7	–	0.990	–	0.019	–	0.982	–	0.85	–	0.92
8	–	0.995	–	0.000	–	1.000	–	1.00	–	1.00
9	–	0.996	–	0.000	–	1.000	–	1.00	–	1.00

**Table 9:** Spikformer validation.

Category	True positive rate		False positive rate		Accuracy		Precision		F1-score	
	IDS-2018	DDoS-2019	IDS-2018	DDoS-2019	IDS-2018	DDoS-2019	IDS-2018	DDoS-2019	IDS-2018	DDoS-2019
0	0.976	0.999	0.011	0.000	0.986	1.000	0.95	1.00	0.96	1.00
1	0.998	0.999	0.000	0.000	1.000	1.000	1.00	1.00	1.00	1.00
2	1.000	1.000	0.000	0.001	1.000	0.999	1.00	0.99	1.00	1.00
3	1.000	1.000	0.000	0.000	1.000	1.000	1.00	1.00	1.00	1.00
4	1.000	0.999	0.000	0.001	1.000	0.999	1.00	0.99	1.00	1.00
5	0.946	0.982	0.005	0.000	0.987	0.998	0.97	1.00	0.96	0.99
6	–	0.994	–	0.000	–	0.999	–	1.00	–	1.00
7	–	0.994	–	0.002	–	0.998	–	0.99	–	0.99
8	–	0.997	–	0.000	–	0.999	–	1.00	–	1.00
9	–	1.000	–	0.000	–	1.000	–	1.00	–	1.00

**Table 10:** CNN validation.

Category	True positive rate		False positive rate		Accuracy		Precision		F1-score	
	IDS-2018	DDoS-2019	IDS-2018	DDoS-2019	IDS-2018	DDoS-2019	IDS-2018	DDoS-2019	IDS-2018	DDoS-2019
0	0.987	0.998	0.011	0.000	0.989	1.000	0.95	1.00	0.97	1.00
1	0.999	1.000	0.000	0.000	1.000	1.000	1.00	1.00	1.00	1.00
2	1.000	1.000	0.000	0.000	1.000	1.000	1.00	1.00	1.00	1.00
3	1.000	1.000	0.000	0.000	1.000	1.000	1.00	1.00	1.00	1.00
4	1.000	0.998	0.000	0.000	1.000	1.000	1.00	1.00	1.00	1.00
5	0.946	0.997	0.003	0.000	0.989	0.999	0.99	1.00	0.97	1.00
6	–	1.000	–	0.000	–	1.000	–	1.00	–	1.00
7	–	0.997	–	0.000	–	0.999	–	1.00	–	1.00
8	–	1.000	–	0.000	–	1.000	–	1.00	–	1.00
9	–	1.000	–	0.000	–	1.000	–	1.00	–	1.00

**Table 11:** Our model validation.

Category	True positive rate		False positive rate		Accuracy		Precision		F1-score	
	IDS-2018	DDoS-2019	IDS-2018	DDoS-2019	IDS-2018	DDoS-2019	IDS-2018	DDoS-2019	IDS-2018	DDoS-2019
0	0.988	1.000	0.012	0.000	0.988	1.000	0.95	1.00	0.97	1.00
1	0.999	1.000	0.000	0.000	1.000	1.000	1.00	1.00	1.00	1.00
2	1.000	1.000	0.000	0.000	1.000	1.000	1.00	1.00	1.00	1.00
3	1.000	1.000	0.000	0.000	1.000	1.000	1.00	1.00	1.00	1.00
4	1.000	0.999	0.000	0.000	1.000	1.000	1.00	1.00	1.00	1.00
5	0.941	0.994	0.003	0.001	0.988	0.999	0.99	1.00	0.96	0.99
6	–	1.000	–	0.000	–	1.000	–	1.00	–	1.00
7	–	0.993	–	0.001	–	0.999	–	0.99	–	0.99
8	–	1.000	–	0.000	–	1.000	–	1.00	–	1.00
9	–	1.000	–	0.000	–	1.000	–	1.00	–	1.00

**Figure 3:**
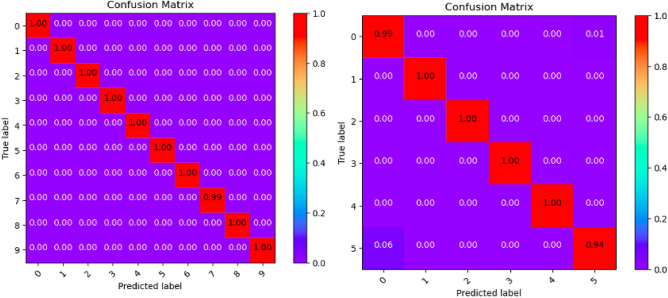
CIFARNet-GLIF validation.

**Figure 4:**
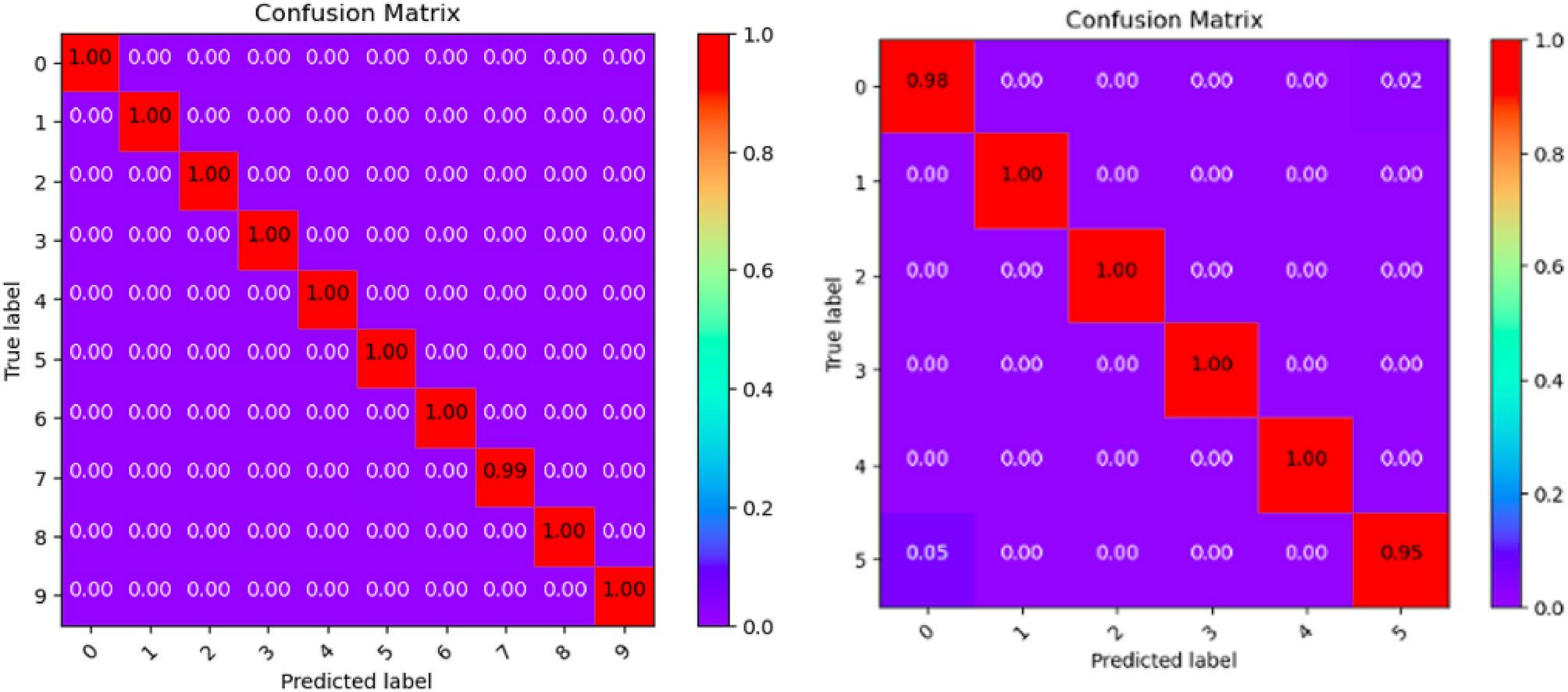
ResNet18-LGLIF validation.

**Figure 5:**
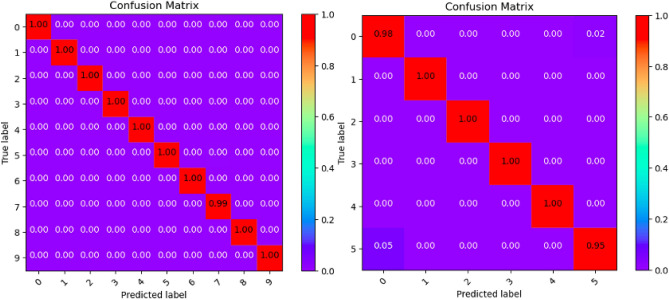
ResNet18-GLIF validation.

**Figure 6:**
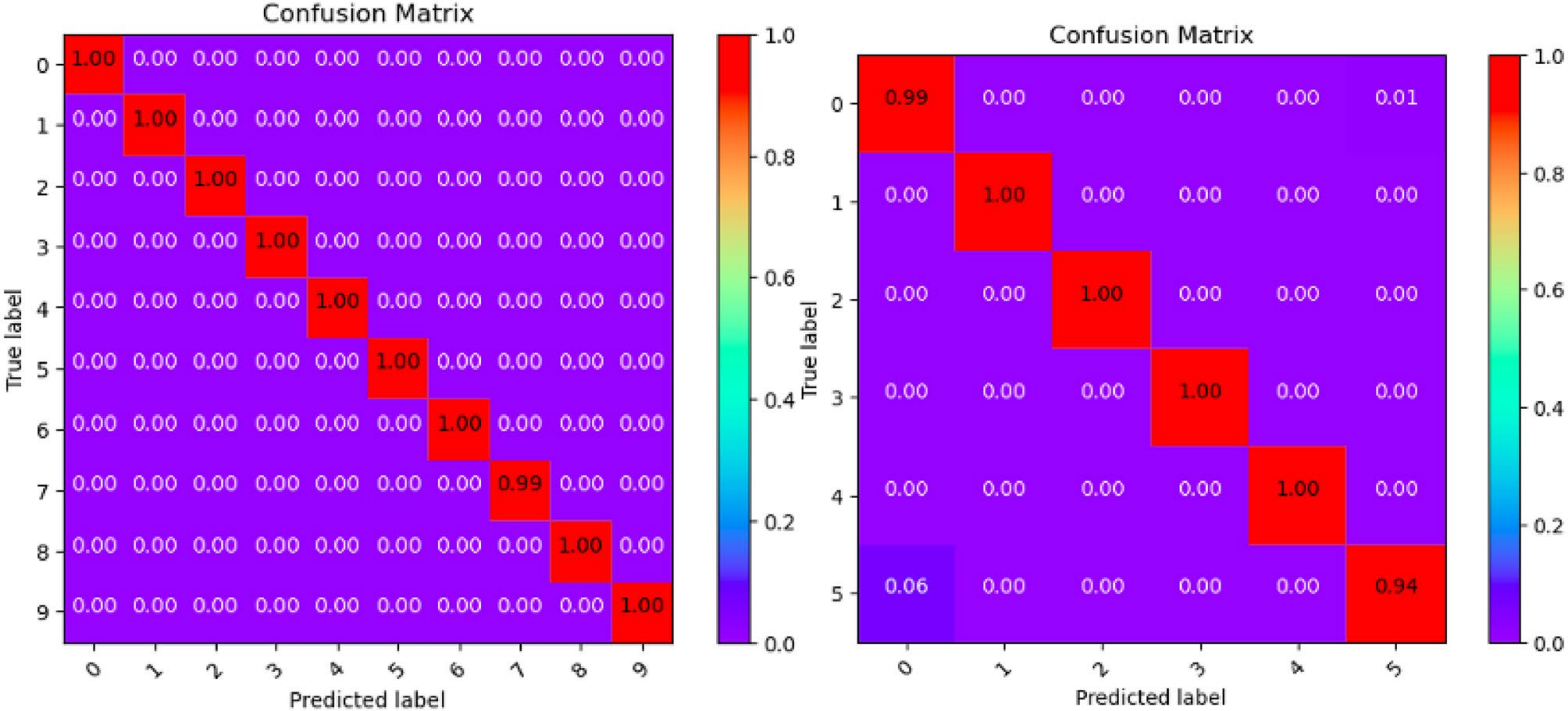
SpikeDHS validation.

**Figure 7:**
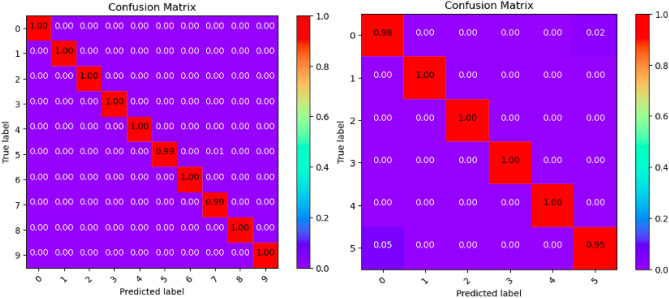
SpikingGCN validation.

**Figure 8:**
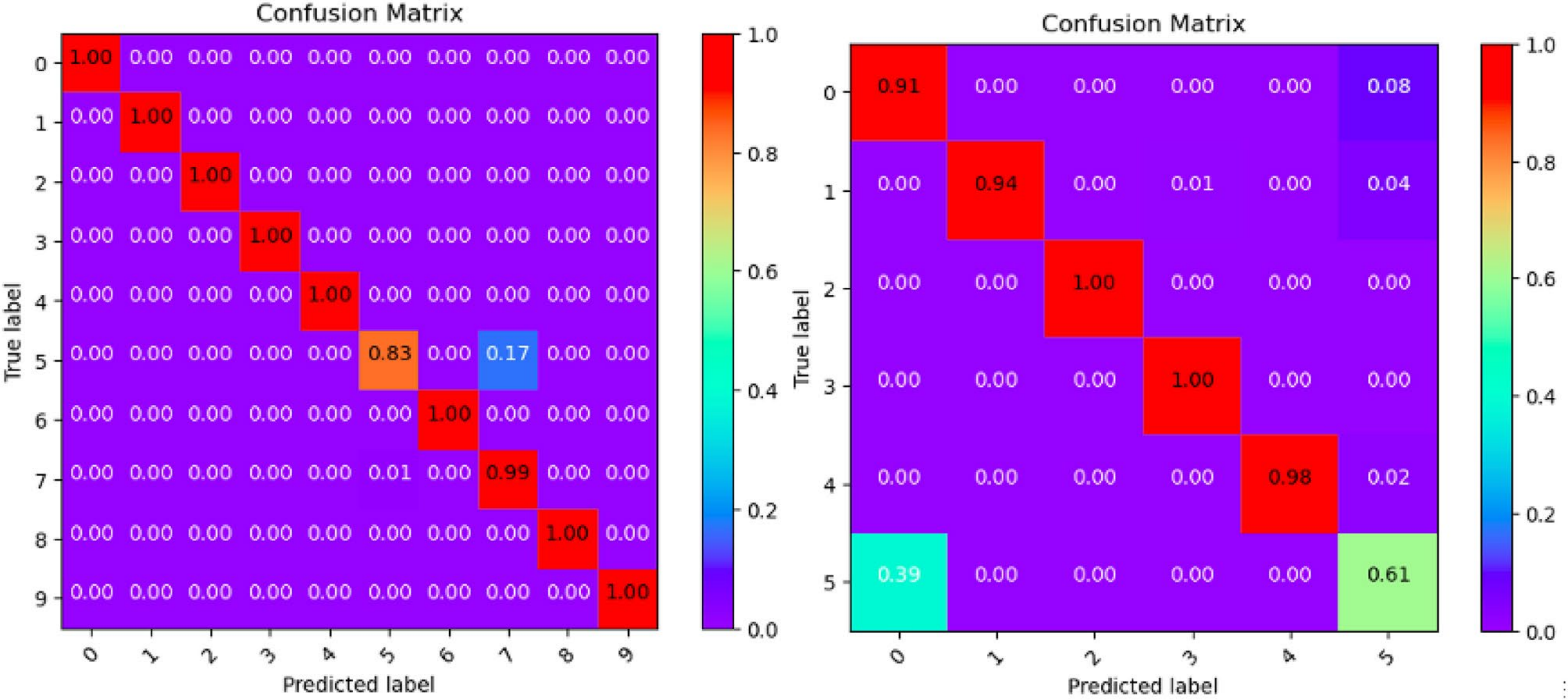
NAS-SNN validation.

**Figure 9:**
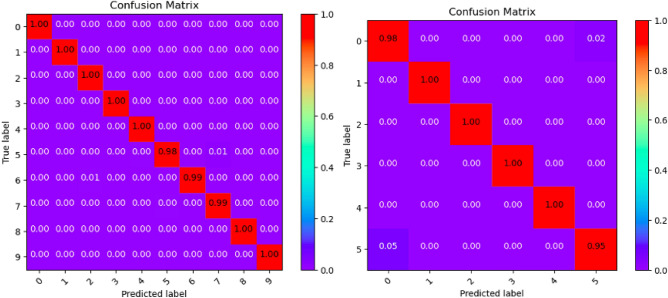
Spikformer validation.

**Figure 10:**
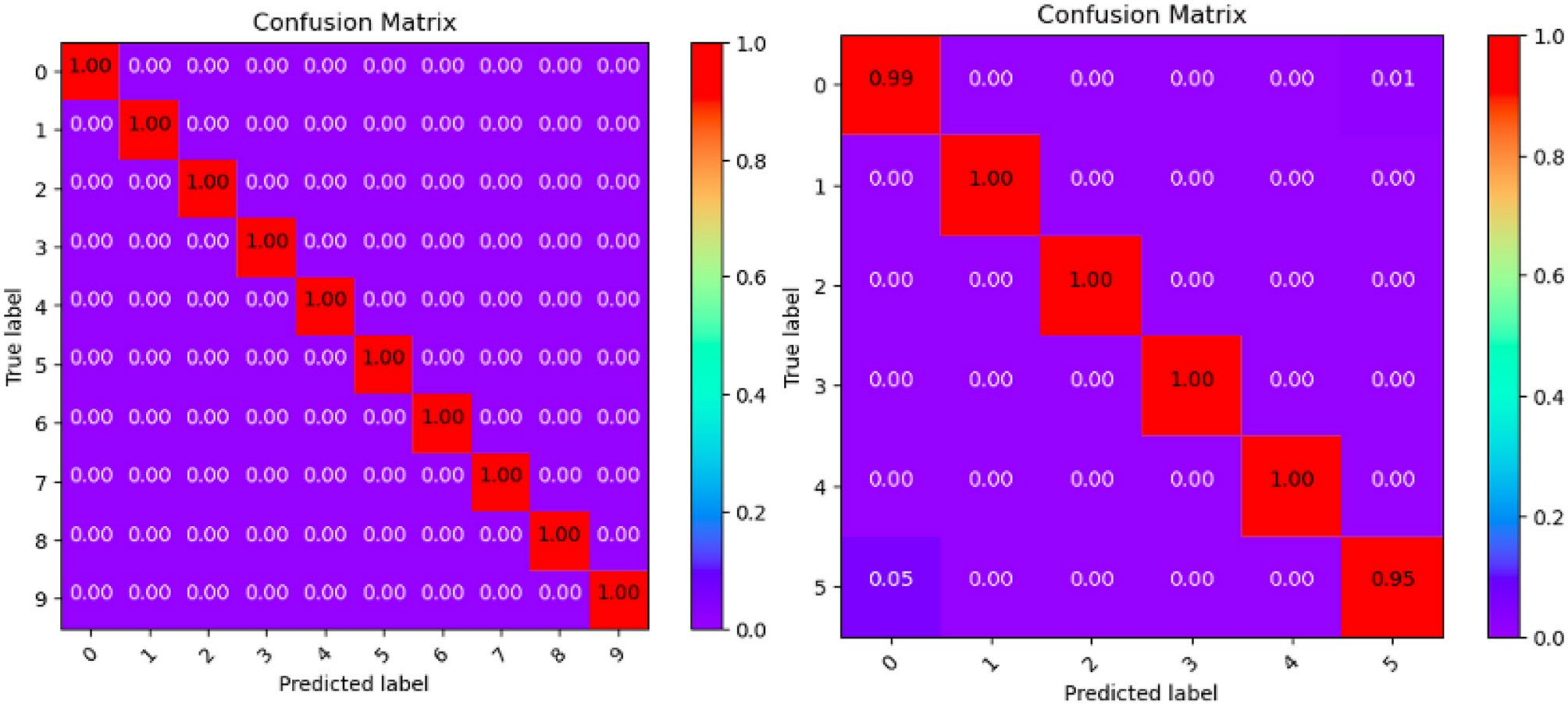
CNN validation.

**Figure 11:**
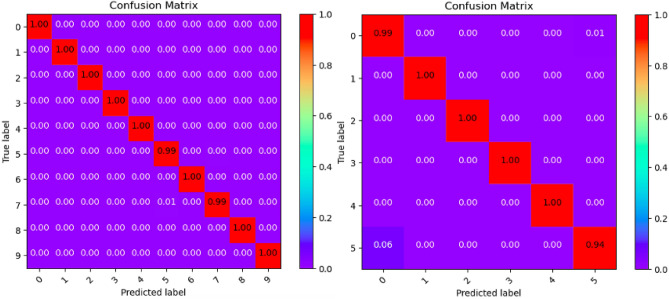
Our model validation.

All the SNN models and CNN structure used in the experiments were published in authoritative publications in recent years. As can be seen from the accuracy results of the experiments, the classical CNN is still slightly higher than most of the SNN models. It shows that in the current research process, SNNs are not yet able to completely replace CNNs in terms of accuracy. At the same time, the model proposed in this paper can achieve the same level of accuracy as these state-of-the-art models. Moreover, it comprehensively outperforms these models in terms of efficiency metrics, which shows that the overall practical capability of the model is excellent. Finally, the models’ ability to cope with novel attacks was tested using DrDoS_LDAP, an attack type that was not present in the training set. The number of samples used for validation was 5000. The detection accuracies of the models trained on the CSE-CIC-IDS2018 dataset and CIC-DDoS2019 dataset are 95.06 and 99.56, respectively. It can be seen that the trained model has a high level of recognition capability even when it encounters unknown types of attacks. They can provide valuable information for security managers.

All the SNN models and CNN structure used in the experiments were published in authoritative publications in recent years. As can be seen from the accuracy results of the experiments, the classical CNN is still slightly higher than most of the SNN models. It shows that in the current research process, SNNs are not yet able to completely replace CNNs in terms of accuracy. Moreover, some SNN models do not dominate over CNNs in the performance metrics for which SNNs are so highly sought after. At the same time, the model proposed in this paper can achieve the same level of accuracy as these state-of-the-art models. Moreover, it comprehensively outperforms these models in terms of efficiency metrics, which shows that the overall practical capability of the model is excellent.

## Conclusions and future work

A total of eight spiking neural network models and one deep convolutional neural network model were used in the experiments. Many IoT devices do not have sufficient computing resources or energy supply due to cost, working environment and other factors. This causes great inconvenience to them in self-protection. Many strategies have been developed to address this challenge through cloud computing services^60–62^, but this inevitably introduces new information security concerns^63^, as well as increasing the burden on networks. The model proposed in this paper is significantly higher in all efficiency metrics, while maintaining a high detection accuracy level. The proposed model also enhances the ability to work properly on devices with limited computational resources and insufficient energy supply. The lack of computing resources and power supply is exactly the dilemma that many IoT devices are currently facing.

Although the proposed model outperforms the existing solutions in terms of efficiency, there is a slight decrease in classification accuracy compared to the most accurate model. As measured by metrics such as classification accuracy, execution efficiency and energy consumption, the spiking neural network models still do not perform as well as deep convolutional neural networks in most cases. The reason for this is primarily due to the complexity of the design of these SNN models, and the presence of this complexity did not lead to an improvement in accuracy. It can be seen that the spiking neural network models still have a lot of work to do to catch up with the classical deep neural networks with regard to overall performance.

In future work, attempts will be made to unite many resource-poor devices and get them to help each other, resulting in a stronger defense system. As a single device has very limited resources, if the resources of many devices are dispatched in an intelligent, efficient, and reasonable fashion, they will be able to address various problems more effectively. A suitable distributed scheduling algorithm will be developed so that, as a whole, these devices with unified resource scheduling will perform better than when they operate independently.

## Data Availability

The datasets used and/or analyzed during the current study available from the corresponding author on reasonable request.
